# Design of study without drugs—a Surinamese school-based drug-prevention program for adolescents

**DOI:** 10.1186/s12889-015-2374-1

**Published:** 2015-10-12

**Authors:** Fariel Ishaak, Nanne Karel de Vries, Kees van der Wolf

**Affiliations:** Faculty of Social Sciences, Anton de Kom University of Suriname, Leysweg, 86 Suriname; Department of Health Promotion, CAPHRI School for Public Health and Primary Care, Maastricht University, Maastricht, The Netherlands; Faculty of Social and Behavioural Sciences, Universiteit van Amsterdam, Amsterdam, The Netherlands; Department of Education, Anton de Kom Universiteit, Tammenga, Suriname

**Keywords:** Adolescents, School prevention program, Educational models, Drug dependence, Intervention mapping protocol

## Abstract

**Background:**

The aim of this study was to design the content and accompanying materials for a school-based program—Study without Drugs—for adolescents in junior secondary schools in Suriname based on the starting points and tasks of the fourth step of the Intervention Mapping protocol (which consists of six steps). A program based on this protocol should include a combination of theory, empirical evidence, and qualitative and quantitative research.

**Methods:**

Two surveys were conducted when designing the program. In Survey I, teachers and students were asked to complete a questionnaire to determine which school year they thought would be most appropriate for implementing a drug-prevention program for adolescents (we completed a similar survey as part of previous research). An attempt was made to identify suitable culturally sensitive elements to include in the program. In Survey II, the same teachers were asked to complete a questionnaire to determine the programs’ scope, sequence, structure, and topics as well as the general didactic principles to serve as a basis for program design. After outlining the program plan, lessons, and materials, we conducted a formative pretest evaluation among teachers, students, and parents. That evaluation included measures related to the program’s attractiveness, comprehensibility, and usefulness. The resulting lessons were presented to the teachers for assessment.

**Results:**

The drug-prevention program we developed comprises 10 activities and lasts 2–2.5 months in an actual school setting. The activities take place during Dutch, biology, physical education, art, religion, and social studies lessons. We based the structure of the lessons in the program on McGuire’s Persuasion Communication Model, which takes into account important didactic principles. Evaluations of the program materials and lesson plans by students, teachers, and parents were mostly positive.

**Conclusion:**

We believe that using the fourth step of the Intervention Mapping protocol to develop a drug-prevention intervention for adolescents has a produced promising, feasible program.

**Electronic supplementary material:**

The online version of this article (doi:10.1186/s12889-015-2374-1) contains supplementary material, which is available to authorized users.

## Background

This article describes the development of Study without Drugs, a drug-prevention program recently implemented in junior secondary schools throughout Suriname. Suriname is located in northeastern South America and has an area of 165,000 km^2^. The population is approximately around 566,000 and very heterogeneous. After around 300 years of colonization—first by England and then the Netherlands—Suriname became an independent republic on November 25, 1975. On November 25, 1980 a coup d’état took place, led by Desi Bouterse. During the period 1980–87, Suriname was often associated with drug trafficking. Democracy returned to the country in 1987.

The Ministry of Education and Community Development is responsible for education in Suriname, which is based on the Dutch education system. In 2004, the Surinamese government announced it would implement education reforms in the country. The central aim of the reforms was to introduce an 11-year basic education system, consisting of integration of pre-primary, primary, and junior secondary schools. The language of instruction is Dutch.

Basic education comprises 2 years of pre-primary school (for children aged 4 and 5 years) and primary school, which lasts 6 years (for children aged 6–11). After completing this education with a final test, pupils are awarded the Certificate of Primary Education. This certificate allows pupils to enter general junior secondary education, which is divided into a general program with a nominal duration of 4 years and a vocational program with a nominal duration of 1–4 years. The vocational program consists of several facets, such as vocation-oriented education. After graduating from general junior secondary education, pupils can move on to general senior secondary education.

General senior secondary education is divided into general education, with a nominal duration of either 3 years (VWO) or 2 years, and vocational education, with a nominal duration of 1–4 years. General senior secondary education diplomas grant admission to higher education (colleges and university). Admission to Anton de Kom University of Suriname is usually granted on the basis of the VWO diploma. Suriname has a binary higher education system. With four faculties and a number of research institutes, Anton de Kom University is the only university in the country. Suriname also has a number of higher professional education institutes as well as an institute offering bachelor’s programs for higher professionals and master’s and postgraduate programs [1].

No evidence-based drug-prevention programs have hitherto been developed and implemented in any schools in Suriname [2]. Study without Drugs is thus the first school-based drug-prevention program in the country; during its development, possible criteria for other effective school-based drug-prevention programs in Suriname were integrated as much as possible This program can serve as a first step toward further development of evidence-based school programs in Suriname We developed the program with the aim of preventing junior secondary school students from abusing alcohol or other drugs; such abuse often starts during adolescence. When adolescents move from elementary to high school, they often experience new stressors, such as increased peer influence, the need to adapt to new educational models and schedules, and greater expectations regarding educational performance. Furthermore, decreased parental supervision likely plays a role in heightened risk of deviant behavior [3–6]. In general, a combination of biological, psychological, and environmental variables determines whether adolescents will start abusing drugs in youth or early adulthood.

### Drug use among Surinamese adolescents

Sponsored by both Suriname and the European Union, the Drug Demand Reduction Program conducted a nationwide Rapid Situation Assessment in 2005, involving 480 people aged >13 years. The results showed that among Surinamese aged 13 years and older, the majority of smokers started before the age of 20; furthermore, 60 % of male and 38 % of female respondents had their first encounter with alcohol before the age of 20 [7]. A 2005 in-person study of 125 junior secondary school students aged 12–16 years by the Drug Demand Reduction Program showed that 20 % of respondents had previously had contact with drug users; the latter included direct family members, friends, fellow school students, and strangers, such as people in the neighborhood and local drug addicts. The students also reported infrequent use of, though relatively easy access to, cocaine. Furthermore, such drugs as the well-known variant *blaka djonko* (a mixture of inferior cocaine and marijuana) were often cheaply offered in, for example, nightlife venues [8].

School surveys conducted in 2004 and 2006 by the Drug Demand Reduction Program found alcohol, tobacco, and marijuana to be the most frequently used drugs among junior secondary school students. In addition, the results from those surveys indicated that students in their first and second years of high school were the likeliest age range for beginning alcohol use and smoking [9, 10].

We suppose that the discrepancies between the students’ self-reporting of drug use in the 2005 in-person study and the two anonymous surveys arose from the students’ reluctance to discuss personal drug use and attitudes openly in an interview setting. Additional findings appear in Table 1.

**Table 1:** Key findings of the 2004, 2005, and 2006 DRPP reports on junior secondary school students’ drug use [6–8]

Drugs used by survey respondents	2004 school survey (nationwide, anonymous questionnaire) *n* = 3569	2005 study (single-site, in-person interview) *n* = 125	2006 school survey (nationwide, anonymous questionnaire *n* = 2206
Alcohol	74.3 %	25 %	63.5 %
Tobacco	35.4 %	29.6 %	35.8 %
Marijuana	43 %	25 %	68 %

*DRPP* drug demand reduction program

There are very few data related to drug use by subpopulation in Suriname. In 2007, the Ministry of Health conducted an investigation among 1413 young people in the urban areas of Latour, Nickerie, Lelydorp and rural areas of Moengo and Brokopondo [11]. In summary, the results were as follows. Alcohol was reportedly the most frequently used drug: 73 % of participants reported having used alcohol at least once in their lives. The proportion of regular users was 6 %, with a clear predominance of males: 10 % of males against 3 % of females. Experimenting with alcohol among participants aged 10–14 years varied from 51 % in Lelydorp to 75 % in Brokopondo. Regular alcohol use was mostly reported by males aged 20–24 years.

About one in five youngsters (21 %) had experimented with tobacco. Among children aged 10–14 years, that proportion varied from 4 % in Latour to 7 % in Brokopondo; among those aged 15–19 years, it varied from 22 % in Nickerie to 29 % in Lelydorp. Regular cigarette smokers accounted for 6 % of all respondents. It is notable that almost all regular smokers were males: 12 % of males against 1 % of females.

Approximately 6 % of the young people in all age-groups reported having used marijuana. Almost all the respondents who had ever tried marijuana were males: 10 % of males and only 1 % of females. Regular use of marijuana was reported by 2 % of all respondents—by 4 % of all males and 0.2 % of all females. Among subjects aged 15–19 years, 6 % had experimented with marijuana while 3 % reported regular use. At 9 %, regular marijuana use was highest among males aged 20–24 years in Brokopondo. About 70 % of the respondents reported having received education about drugs.

The Comparative Analysis of Student Drug Use in Caribbean Countries is a summary of school surveys conducted in 12 countries between 2005 and 2007 [12]. The population targeted for that survey included students at secondary-level (or high) schools in forms 2, 4, and 6 (broadly equivalent to grades 8, 10, and 12 in the US school system). The respondents were generally aged 13, 15, and 17 years. The total sample amounted to 38,534 students. Drug use was measured in terms of lifetime, previous year’s, and previous month’s consumption. Tables 2, 3 and 4 present the prevalence of alcohol, tobacco, and marijuana usage.

**Table 2:** Prevalence of alcohol use

Country	Lifetime prevalence	Past-year prevalence	Past-month prevalence
Antigua and Barbuda	71.10	49.20	31.32
Barbados	75.60	54.73	34.57
Dominica	80.18	66.05	51.58
Grenada	80.77	58.94	39.55
Guyana	61.02	46.40	36.79
Haiti	47.72	26.91	18.27
Jamaica	65.83	47.03	33.38
St Kitts and Nevis	64.77	45.41	30.04
St Lucia	86.20	73.64	61.95
St Vincent and the Grenadines	65.34	46.38	33.21
Suriname	61.15	44.78	31.80
Trinidad and Tobago	82.08	66.60	48.23
Average	68.90	51.00	36.70

**Table 3:** Prevalence of tobacco use

Country	Lifetime prevalence	Past-year prevalence	Past-month prevalence
Antigua and Barbuda	17.31	4.47	1.79
Barbados	21.46	8.25	3.66
Dominica	30.69	13.22	7.76
Grenada	34.53	10.75	5.45
Guyana	17.66	6.10	4.13
Haiti	9.20	3.25	1.49
Jamaica	24.61	8.37	4.44
St Kitts and Nevis	11.82	4.00	1.98
St Lucia	27.84	11.31	5.97
St Vincent and the Grenadines	21.01	5.43	2.66
Suriname	33.06	14.15	7.15
Trinidad and Tobago	28.86	11.36	5.61
Average	25.60	9.45	4.94

**Table 4:** Prevalence of marijuana use

Country	Lifetime prevalence	Past-year prevalence	Past-month prevalence
Antigua and Barbuda	23.94	12.88	8.05
Barbados	18.97	11.42	6.88
Dominica	29.54	17.47	11.62
Grenada	25.43	14.44	7.75
Guyana	12.12	6.87	3.97
Haiti	2.20	1.05	0.63
Jamaica	21.56	12.04	7.06
St Kitts and Nevis	24.14	12.85	6.94
St Lucia	26.51	16.49	9.46
St Vincent and the Grenadines	20.13	12.92	5.82
Suriname	5.53	3.29	2.02
Trinidad and Tobago	12.09	6.44	2.70
Average	17.03	9.76	5.39

Alcohol use in each country appears in Table 2. The overall average lifetime prevalence was 68.90 %, ranging from 47.72 % in Haiti to 86.20 % in St Lucia. With the exception of Suriname, Guyana, Jamaica, St Kitts and Nevis, and St Vincent and the Grenadines, all other countries reported lifetime prevalence well above the overall average (more than 70 %). The prevalence of alcohol use over the past year ranged from 26.91 % in Haiti to 73.64 % in St Lucia. Haiti’s results almost make it an outlier since the country with the second-lowest previous year’s consumption was Suriname, at 44.78 %. The results for that group of countries can be easily classified into low (under 40 %), average (40–60 %), and high (over 60 %) prevalence groups. Only Haiti could be included in the low group; Dominica, St Lucia, and Trinidad and Tobago would be classified as high-prevalence countries in terms of previous year’s consumption. Consumption over the previous year was similarly high for Suriname (14.15 %), Dominica (13.22 %), Trinidad and Tobago (11.36 %), and Grenada (10.75 %). The overall average previous year’s consumption was 9.45 %; with the exception of St Lucia (11.31 %), most other countries reported low prevalence rates. The lowest consumption rates were reported by Haiti (3.25 %), St Kitts and Nevis (4 %), and Antigua and Barbuda (4.47 %). The low previous year’s consumption rates in those countries were as low as the overall average previous month’s consumption.

As indicated in Table 3, smoking during the previous month (current smokers) showed a relatively low prevalence among students in the 12 countries surveyed. Dominica (7.76 %), Suriname (7.15 %), St. Lucia (5.97 %), and Grenada (5.45 %) were the only countries where the previous month’s prevalence rates were greater than 5 %. The lowest prevalence was reported by Haiti (1.49 %) and St Kitts and Nevis (1.98 %).

The lifetime, previous year’s, and previous month’s prevalence of marijuana use in the countries surveyed appear in Table 4. There are large variations in the prevalence rates by country, ranging from Haiti (2.2 %, 1.05 %, and 0.63 % for lifetime, previous year, and previous month, respectively) to Dominica (29.58, 17.47, and 11.62 % for lifetime, previous year, and previous month, respectively). Almost 30 % of students in Dominica have tried marijuana at least once in their lives; 17.5 % had used it in the past year and about 12 % currently used the drug. St Lucia and Grenada were the only other countries where over 25 % of students had experimented with marijuana at some point in their lives. Similarly for use over the previous year, Dominica and St Lucia evidenced the highest rates (over 15 %); Grenada was just below 15 %. By contrast, Haiti and Suriname both showed a very low prevalence over the past year of under 5 %. Trinidad and Tobago was also at the lower end of the scale, with a reported prevalence of just over 5 % in the past year.

From the above description and Tables 1, 2, 3 and 4, it may be concluded that the rate of drug use among adolescents in Suriname is not alarmingly high for the region, and the data appear to fit well within the norms for other countries worldwide. However, preventive measures are necessary to keep the observed tendencies under control.

### Theoretical basis of a program for preventing adolescent drug use

The literature suggests that the structure and contents of an information program, i.e., a program aimed at influencing personal determinants, can be based on McGuire’s Persuasion Communication Model [13]. According to this model, the process of behavioral change goes through seven phases: (a) gaining attention for and understanding information; (b) gaining knowledge; (c) perceiving risk; (d) building up one’s attitude; (e) incorporating supportive social influences; (f) creating self-efficacy; and (g) maintaining behavioral change. These phases are subsequently targeted in the developed information program. Furthermore, some studies [14, 15] have determined that such a program has to include, among other aspects, the following: elements that encourage participation; explicit links between the program and other subject matter; a variety of teaching methodologies; and a didactic working method as well as interim and final evaluations.

School prevention programs based on McGuire’s Persuasion Communication Model [13] include components to help students develop both personal and social aspects. Through this approach, students may be able to recognize influences (such as advertising and peer influence) that encourage the use of harmful substances and thereby help build resilience to cope with such pressure. These programs focus on the development of the following skills: stress management goal setting; communication; and assertiveness. Interactive methods—where students have an active role in information sharing, e.g., role playing and discussion—are more effective than simply passively receiving information [16–26].

In addition to knowledge, life skills, such as communication and resilience, are also practiced as part of school interventions. Studies have shown that the effects of interactive school interventions are minimal—especially in the shorter term (such investigations are typically conducted soon after the intervention is completed). It may be possible, however, that the effects exert a significant impact because of the wide range of school interventions [19].

Furthermore, it may be noted that if interventions focus on the school’s overall social environment, they appear to be more effective [20, 21, 27]. School interventions also prove more effective when parents and education professionals are involved. Such a comprehensive approach is desirable toward making school prevention programs more effective: it is necessary that the message behind the intervention be supported both at home and in the school environment [19, 23, 26].

### Cultural sensitivity of health and prevention programs

Studies have demonstrated that school-based drugs-prevention programs—as a type of health-promotion program—are culturally sensitive. Cultural sensitivity is defined by two dimensions—surface structures and deep structures. Surface structures involve matching intervention materials and messages to observable, superficial characteristics of a target population. Surface structures may involve using people, places, language, music, food, locations, and clothing familiar to and preferred by the target audience. Surface structures refer to how well interventions fit within a specific culture. Deep structures involve incorporating the cultural, social, historical, environmental, and psychological forces that influence the target health behavior in the target population. Whereas surface structures generally increase the receptivity or acceptance of messages, deep structures convey salience [28].

The manner in which people experience various aspects of preventing substance abuse and treatment intervention is affected by culture [29, 30]. To respond effectively to the unique needs of different target groups, it is necessary to execute substance abuse interventions in a culturally competent manner [31, 32]. Interventions have to be sensitive to the cultural values of a particular population while also addressing common etiological factors of substance abuse. Cultural competence involves understanding and appreciating the important role that cultural differences play in people’s health beliefs and behavior [33–35]. Creating culturally competent services is not an easy process. Nevertheless, substance abuse interventions can ensure cultural representation by including family members and other supporting figures as well as recruiting community members to participate in program planning, development, and delivery. This may involve using people, places, language, music, food, locations, and clothing familiar to and preferred by the target audience.

If the program reflects the perceptions of a specific target group, it will increase the group’s receptivity to its underlying prevention and intervention messages. In summary, the development and integration of a culturally competent plan in substance abuse interventions is a key strategy toward effectively addressing the prevention and treatment needs of target groups.

**Fig. 1:**
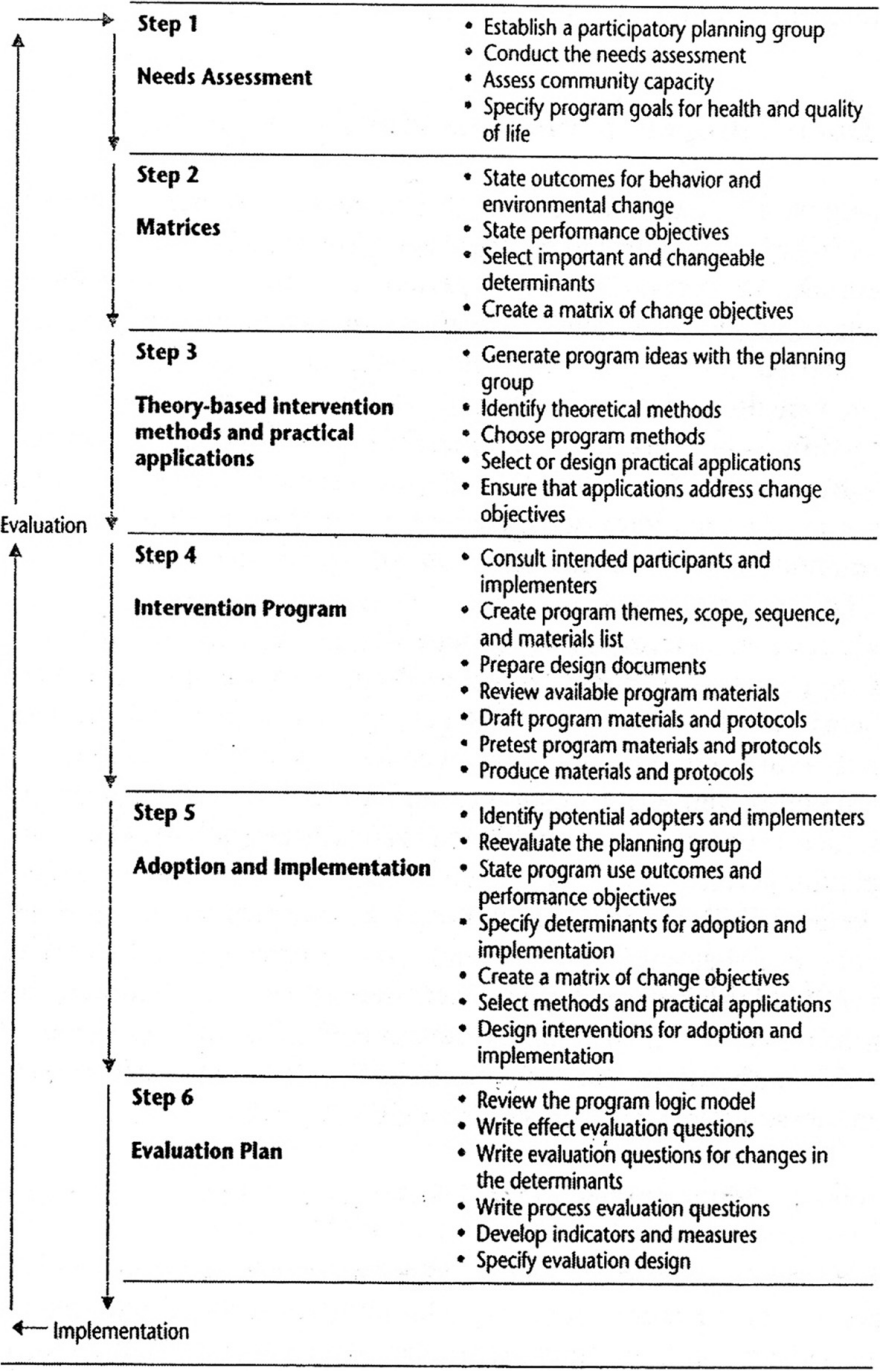
Intervention mapping (Bartholomew, Parcel, Kok & Gottlieb, 2011)

In developing the content, program overview, and supporting material for Study without Drugs, we sought to implement the above-mentioned methodologies during the fourth step using an Intervention Mapping (IM) protocol (Fig. 1). The IM protocol is a six-step protocol designed to ensure that the prevention program is based on a combination of theory, empirical evidence, and qualitative and quantitative research. This protocol lends itself well as a tool for developing, implementing, and evaluating a theoretical and evidence-based school-based drugs-prevention program that can be categorized as a health-promotion program.

Involving stakeholders and participants is central to this program. Such programs for adolescents have to take into account the varied biological, psychological, and environmental factors related to their decision-making behavior. In addition, the IM protocol requires pretesting program strategies and materials for executors and receivers (Fig. 1 and Additional file 1).

In Step 4, there is a description of the program plan and the way in which program components are to be developed. This step also involves the pretesting of program strategies and materials intended for use by the executors and receivers. The IM protocol has not yet been tested in Suriname; however, with its scientific approach in gathering data about young people in general, this protocol shows good potential for developing a program for young people in that country.

This article addresses two questions: at what junior secondary school grade should a school-based drug-prevention program be offered for maximum efficacy? What should the contents of that program be? With Study without Drugs, we assert that the program should incorporate an IM methodology and concomitant evaluations by teachers, parents, and students.

## Methods

In developing the drug-prevention program, we successively applied the following methods: (1) surveys—Surveys I and Survey II; (2) group interviews among teachers, students, and parents for pretesting material using the plus–minus method; and (3) utilizing a questionnaire to assess the lessons developed.

### Surveys (I and II)

For this study, we developed and subsequently administered two surveys at a junior secondary school. The aim with Survey I was to determine the extent to which the participants would reconfirm a previously suggested level—namely the second year of junior secondary school—as the appropriate grade for introducing the drug-prevention program. The objective with Survey I was not to go into details of cultural sensitivity. Respondents were asked therefore only to create a list of culturally sensitive issues or components that questionnaire respondents would like to see included within this type of program.

The questionnaires, including an instruction sheet, were distributed to the respondents via the school principal. The questionnaires were collected 1 day later. A total of 694 students and 236 teachers participated in Survey I. An overview of the respondents’ demographic details is provided in Table 5.

**Table 5:** Demographics of survey I respondents for the IM-based drug-prevention program

Category	Teachers	Students
Response rate	95.3 % (225/236)	694 (100 %)
Average age	39 years	15 years (range, 13–16)
Gender	80 % female, 20 % male	66 % female, 34 % male
Educational experience	71.2 % working in junior secondary schools in general education; 23.3 % in junior vocational education; 5.5 % in junior technical education	70.2 % in junior secondary schools; 25.4 % in junior vocational education; 4.3 % in junior technical education
	53 % with >10 years’ experience in education	--

For Survey II, the teachers who participated in the first survey were approached again. A number off 225 participants confirmed that they would also take part in the follow-up survey. This time, the teachers were asked about the scope and sequence of program elements; they were also asked which subjects they considered would be most appropriate for offering drug-prevention lessons. We checked whether using lessons as the basis for the program would concur with McGuire’s Persuasion Communication Model [13] and to what extent general and specific didactic principles should be taken into account when developing the material. Teachers where informed in an special brochure about the general structure of the McGuire’s model, which is widely used and effective in developing health programs. Therefore, as part of the survey questionnaire, we sought to assess whether teachers thought they would find value in the general integration of the structure of the model.

### Group interviews for pretest using plus–minus method

We tested the study materials using the plus–minus method and group interviews. The plus–minus method is simple to execute and can yield sufficient relevant information in a reasonably short time [36–38]. The participants were 47 junior high students (average age, 16 years; 61.7 % male), 20 teachers (average age, 43 years; 80 % female; 66 % with >10 years of experience), and 25 parents with a child attending junior secondary school (average age, 44 years; 15 mothers, 10 fathers). All respondents received information explaining the program at appropriate stages. The students and parents took a pretest to measure their knowledge at the start of the intervention. Thereafter, they received posters, a brochure, and a letter—written for either students or parents—containing detailed information, an informational handbook about drugs, an informational DVD, and a classroom presentation. They were also asked to watch a film about the program. They then performed a post-test to measure the efficacy of the intervention.

In addition to the post-test, these students and parents were required to assign a plus or minus sign as a score for each program element. After scoring each of these elements, respondents had the opportunity to report their impressions of the materials and their effectiveness in group interviews. The participants were asked in groups to explain their plus and minus responses, their ideas for improvement, and positive assessments regarding the material. On a flip chart visible to all participants, researchers wrote down the criteria for determining the attractiveness, comprehensibility, and usefulness of the program materials as indicated by the respondents.

### Lesson assessment by teachers

The same group of pretest teachers (*n* = 20) was asked to assess the lesson plans developed for their subjects by means of a questionnaire. The questionnaire focused on scoring the following 12 criteria: necessity to train teachers; compatibility with the program; compatibility with students’ interests; structure according to didactic principles; learning objectives clearly formulated; textually correct; incorporation of interim evaluation; incorporation of final, summative evaluation; opportunities for modification; performable tasks; appropriate teaching methods; and appropriate visual aids. Scoring of the criteria was achieved using a three-point scale (agree, disagree, or no opinion).

### Ethical considerations

Ethical clearance was obtained from the Ministry of Education, particularly its ethics committee and the Department of Inspection for Continuing Education at Junior Levels. A letter from the ministry was sent to the participating schools, stating that the ministry gave its consent for developing the program and that it sought cooperation from school management and staff. All school managers and teachers approached by the ministry willingly agreed to participate.

With regard to obtaining the permission and cooperation of students and parents, each participating student received a letter asking for their parents’ permission to take part. All parents gave permission for their children to participate in Survey I and in the pretest. Parents were asked by the school to take part in the pretest of the study material. All individuals approached signaled their willingness to participate.

## Results

### Surveys

Survey I determined that 185 (82.2 %) teachers and 550 (79.2 %) students stated that the second year of junior secondary school (students predominantly aged 12–14) was the ideal time to undertake a drug-prevention program. Though they were not directly asked, it became apparent from the responses that both teachers and students (80 %–95 %) wished to see the following culturally sensitive aspects included in a school-based drug-prevention program: consideration of the language use and mindset of young people toward drug use and challenging behavior in general; using the dance styles of peers; and receiving input from parents, local sports personalities, and popular entertainers, such as singers, comedians, and actors.

In Survey II, 75.8 % (171/225) of teachers reported that a duration of 2–2.5 months would be appropriate for a school-oriented drug-prevention program. Furthermore, the majority of teachers (71.9 %) were in favor of offering 6–10 prevention lessons and the same number of activities. The teachers also rated the following subjects as being the most appropriate for program integration: Dutch (65 %); biology (57.6 %); physical education and art (40.6 % each); and other, e.g., religion and social studies (23.5 %). The majority of teachers (65 %) agreed that the structure of the prevention program lessons should be based on McGuire’s Persuasion Communication Model [13] and most thought that didactic principles should be incorporated. All responding teachers reported that the program’s lesson plans would need to include ways of ensuring student participation and respect for the opinions of others.

As an example of the application of McGuire’s Persuasion Communication Model, each lesson was introduced by an activity that aimed to attract the students’ attention or interest in the theme of the lesson and to determine their previous knowledge. Subsequently, the core of each lesson involved providing knowledge on a particular topic, e.g., the availability of alcoholic drinks on the market and the consequences of having excessive amounts of alcohol in the blood. In the next phase, the respondents received questions and instructions, such as about their own position or attitudes toward excessive alcohol use and how they would explain their position to others. Later lessons were devoted to identifying organizations or individuals that could provide support in solving related problems. These took the form of asking students to name aid agencies that could provide assistance with these problems. In the final evaluation phase of lessons, the focus was on maintaining the desired anti-drug behavior, e.g., by the students writing phrases, slogans, or short sentences, indicating how they would continue to have a positive attitude to drug abuse.

### Program plan: structure, content, accompanying materials

**Fig. 2:**
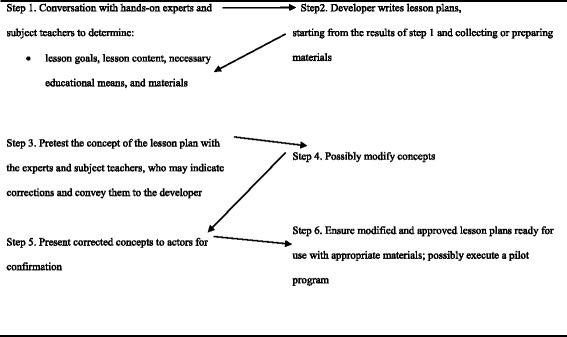
Overview of steps required to develop the studied drug-prevention program’s lesson plans

Based on the results of Surveys I and II and a literature review, we developed the program for Study without Drugs, which consisted of a brochure, introductory letters to parents and students, a Microsoft PowerPoint presentation, seven lesson plans (Table 6), an informational drug handbook, and a poster. Existing materials considered suitable for use in the program were also included, among which were photographs and the films *Maria Full of Grace* and *Rambo* (both on DVD). *Maria Full of Grace* is a Latin American movie that focuses on the perils of drug trafficking; *Rambo* is a Suriname based film that has the same name as the famous Rambo film, that is written and set in Suriname and presents the story of a junior secondary school student who abuses and sells drugs. We also created a series of documents, detailing all aspects of the program, such as potential users and program order. Figure 2 provides an overview of the steps involved in developing the program’s lesson plans. Our school-based drug-prevention program plan consists of seven lessons, one preparatory activity, one informative activity, and two evaluation stages (Table 6).

**Table 6:** Overview of the program plan

Order	Activity	Aim	Means, materials
1	Conduct pretest on drugs	Measure student knowledge and attitudes about drugs	Questionnaire
	Duration: 30 min		
2	Interpersonal information activities	Ask for student’s attention during drug-prevention program and offer information about the program.	Folder, PowerPoint presentation of sample images of drugs
	Duration: 2 h (activities performed outside class)		
3	Subject: biology	- Categorize discussed drinks	Lesson plan, drug handbook, photos
	Consequences of alcohol, tobacco, marijuana, and cocaine use	- State consequences and hold discussion	
	Duration: 40 min		
4	Subject: physical education	- Educational conversation about ethics, including discussion of four consequences of drug use in sports	Lesson plan, photos (for example, steroid use)
	Sports and drugs (steroids)		
	Duration: 40 min		
		- Measures to protect against drug use	
5	Subject: Dutch	- Put events in the correct order and discuss them	Lesson plan, photos, DVD of film (*Maria Full of Grace*), blackboard, chalk
	Discussion about the consequences of drug trafficking and swallowing cocaine pellets		
		- Indicate consequences of swallowing pellets	
	Duration: 80 min		
6	Subject: social studies	- Put events in the correct order	Lesson plan, photos, DVD of play (*Rambo*), blackboard, chalk
	Broadcast of play with anti-drug message	- Arrive at clear understanding of drug use among young people	
	Duration: 60 min	- Indicate causes and consequences that lead to drug use among students	
		- Consider protective measures	
7	Subject: Dutch	- Write and present a slogan	Lesson plan, drug handbook, photos, blackboard, chalk
	Write an anti-drug slogan		
	Duration: 40 min		
8	Subject: art	- Design a poster	Lesson plan, folders, poster, drawing materials, drug handbook, chalk
	Posters with anti-drug message		
	Duration: 40 min		
9	Subject: Dutch	- Initiate conversation through a game on drugs	Lesson plan, game materials
	Game about drugs		
	Duration: 40 min	- Create a loving relationship in a game	
10	Post-test	Measure student knowledge, skills, and attitudes at end of program	Test
	Duration: 30 min (activities performed outside class)

### Pretest results with the plus–minus method

The students (*n* = 47), teachers (*n* = 20), and parents (*n* = 25) who participated in the pretest were asked to assess the acceptability and feasibility of the developed program. The design of the materials was rated positively by 96 % of the students, and all student respondents agreed that the criteria selected for the program were useful. Potential areas of improvement noted by the students related to the poster, the comprehensibility of the brochure, and one of the films (*Maria Full of Grace* was in Spanish with Dutch subtitles).

Pretest results showed that all the teachers assessed the material positively with regard to design, comprehension, and utility. With respect to comprehension, 2 of the teachers gave a minus score for the information book, pretest, posttest, and student letter. Concerning program utility, one teacher gave a minus score for the information book, pretest, post-test, and student letter. Teacher remarks about improvements, e.g., that the language should be clearer and the poster photos larger, led to minor changes in the program materials. All parents responded positively to the design, comprehension, and utility of the program materials. It should be noted with respect to the material presented in Fig. 1 that the comments of students were taken into consideration during the actual production of materials, such as by professional designers, in terms of folder and poster size. Additionally, a language expert and a specialist that is pedagogue also need to be involved to determine the proper language use and visuals appropriate for young people. Some verbal comments made by students appear in Table 7.

**Table 7:** Student statements during the group interview

Positive remarks	Points to improve
● “I’m learning a lot.”	● “The pretest picture is rather small.”
● “Attractive folder and poster”	● “The poster is attractive, but it could be larger?” (2)
● “The film and DVD are OK.”	● “I don’t understand everything in the folder.” (3)
● “The booklet really has nice info.”	● “Can’t the film be in English?” (2)
● “I will always use this book during lessons.”	● Why is it a Spanish film? I can’t follow it.” (1)
● “The letter is good for my parents.”	● “I can't read the translation. It’s too fast.” (1)
● “Much varied material”	● “The Spanish film is difficult to understand, even with English subtitles.” (2)
● “Will this class also participate in the program? I really want to do it.”	
● “Such a funny DVD and you learn something from it. The film is also nice.”	

### Teacher assessment of lesson plans

The teachers were asked to make a separate assessment of the lesson plans: 11 of the 20 teachers indicated that pre-lesson training was not necessary; 8 of the 20 (mostly teachers in physical education, Dutch, social studies, and art) indicated that training was needed. Regarding improvement, the teachers stated that language-specific aspects of the lesson plans deserved attention to ensure easy comprehension by both students and teachers: improvement of language use needed to be adapted to the students’ level. The seven phases of the McGuire Persuasion Communication Model in lesson structure could be understood by 11 of the 20 teachers. All the teachers, regardless of their subject, evaluated the lessons positively.

## Discussion

It is evident from the literature that the most appropriate age-group for a program such as Study without Drugs is the second year of junior secondary school, i.e., 12–15 years of age. It is argued that the greatest risk of drug use occurs during this important transition period in children’s lives. This transition includes significant changes in physical development (puberty), social situation, and movement between grade levels; at that stage, children experience increased vulnerability to peer influence and other risk factors that can lead to deviant behavior [3, 4, 6, 39–41].

Surveys and studies conducted in Suriname have shown that Surinamese students are most likely to try alcohol, tobacco, and marijuana for the first time during adolescence, between the ages of 13 and 15 [7-9-10]. Those findings and other reports detail adolescent drug use and later dependency [16, 40–42], which supports our decision to focus our school-based drug-prevention program on students aged 12–15 years. We concluded that students in the first and second years of junior secondary school are at increased risk of engaging in drug use. Thus, they can be seen as the ideal target group for a school-based program.

The reactions of teachers regarding the scale and construction of the program and its structure are in line with the results of studies into the factors that determine the efficacy of school-based prevention projects. Dusenbury et al. [27] identified several key components related to this effectiveness: the program should have a solid theoretical basis and be founded on academic research; the program should be spread appropriately through the school year; and it should have adequate follow-up sessions. That is to prevent the positive effects of the program weakening with time. Tobler et al. [23] concluded that drug-prevention programs yield more positive outcomes when they are more restricted, smaller, and focused. We therefore aimed to include those elements in Study without Drugs.

Methodologically, the composition of the sample that participated in the pretest of the developed materials may have influenced the outcome since all the participants were volunteers. The subjects also performed under time pressure: limited time was available for the pretest of the developed material. That may have led to socially desirable replies because the students, teachers, and parents may have felt compelled to complete the pretest in a timely manner.

After the pretest, an interview was conducted, in which each respondent could explain their scoring. In retrospect, that may have influenced their replies for reasons of accountability. First, the presence of the interviewer may have influenced the outcome of the answers. Second, the respondents who participated in the pretest were chosen by the respective school management teams. Those respondents may thus possibly have felt obliged to give positive replies to questions. Moreover, the sample was an exploratory sample: it was just a small group of parents and teachers who were approached for the pretest. Therefore, the sample was not completely representative.

Regarding the cultural sensitivity of the program content, we made an attempt during Survey I to inquire which cultural elements should be adopted in the program. However, that attempt to identify cultural elements may be considered rather superficial. In reproducible research, it is generally recommended that greater attention be paid to issues of cultural sensitivity. Furthermore, we followed one of the basic principles of IM—comprehensive involvement of stakeholders by involving students, teachers, and parents during the survey and pretesting.

## Conclusions

We learned a number of lessons while developing Study with Drugs. For example, regarding responses, it is very important to take into account the opinions of all parties involved: those opinions relate to the suitability and usefulness of the materials being developed. Furthermore, it is necessary to bear in mind that developing program content and accompanying materials is not an easy task since various aspects have to be considered, e.g., sufficient finances and knowledge of technical aspects of producing such materials as posters and brochures.

Program developers need to be informed about those factors that help determine whether lessons and material may be effective, e.g., whether they incorporate the language and ideas of contemporary youth regarding such subjects as drugs. It is advisable that developers familiarize themselves with existing effective programs and materials and have a planning and design team available before embarking on an ambitious task like developing a program plan and accompanying materials. We used the fourth step of the IM (basing the development of our drug-prevention program on existing evidence and theories) and followed that protocol in implementing the pretests and initial pilot evaluations; that allowed us to design the content of the supporting material for a school-based drug-prevention program in Suriname. Further research in the form of test implementation is necessary to provide insight into the program’s effectiveness. Such test implementation would allow an experiment and control group to be compared with each other. With respect to composition of the groups, VAB-matched respondents should be used and the principles of randomized controlled trails adopted as far as possible. Both the process and effect of evaluation need to be taken into account.

## Additional file

Additional file 1:
**Intervention mapping.** (DOC 1146 kb)
