# Supplementary material for: Design of study without drugs—a Surinamese school-based drug-prevention program for adolescents
Source: BMC Public Health. 2015 Oct 12;15:1046. doi: 10.1186/s12889-015-2374-1 (PMC4603754; doi:10.1186/s12889-015-2374-1)
Supplement: Additional file 1: — Intervention mapping. (DOC 1146 kb) [file 12889_2015_2374_MOESM1_ESM.doc]

**Appendix INTERVENTION MAPPING**

The six steps of IM are: needs assessment (Step 1): definition of program and change goals; (Step 2) selection methods based on theory and practical strategies; (Step 3) production of program components; (Step 4) anticipation on program adoption; (Step 5) and anticipation of implementation of the process and impact assessment of the program (Step 6)

**Step 1 The needs assessment**

Before an actual design of intervention, health problems, related behavior and the environment, together with the related factors which determine risk populations, should be assessed. The needs assessment can be carried out in accordance with the Predisposing, Reinforcing, Enabling Constructs in Education, Diagnosis and Evaluation (PRECEDE) model. The product of this first step is a description of a health problem, the impact on the quality of life, behavioral and environmental causes and determinants of behavioral and environmental causes.

**Step 2 The definition (formation/fixing) of program and change goals**

In Step 2 general program goals are formulated, which in turn can be specified in behavior goals. The product of Step 2 is a collection matrices in which behavior goals (performance objectives) are combined with selected personal and external factors.

**Step 3 Choice of theory-based methods and strategies**

The program developer chooses theory-based methods and strategies in order to bring about change in a responsible manner in the health habits of persons and related small groups or in organizational and social factors, which have an impact on environment and behavior.

**Step 4 Program design and production of program (intervention program)**

In Step 4 a description of the program plan (intervention program) is given and the way in which the program components should be developed. It also requires the pre-testing of program strategies and materials which are intended for the supervisor and receiver.

**Step 5 Adoption and implementation**

The focus of Step 5 is adoption and implementation of the program. Within this step, matrices are described with behavioral objectives regarding adoption and implementation. After that, these objectives are then operationalized by the use of methods and strategies for the outline of a theory-based plan for adoption and implementation. The product of Step 5 is a detailed plan for the establishment of adoption and implementation.

**Step 6 Anticipation of process and impact assessment**

In the last step (Step 6), an evaluation plan is made, which focuses on impact and process evaluation of the program. An assessment model, or evaluation plan is then developed.


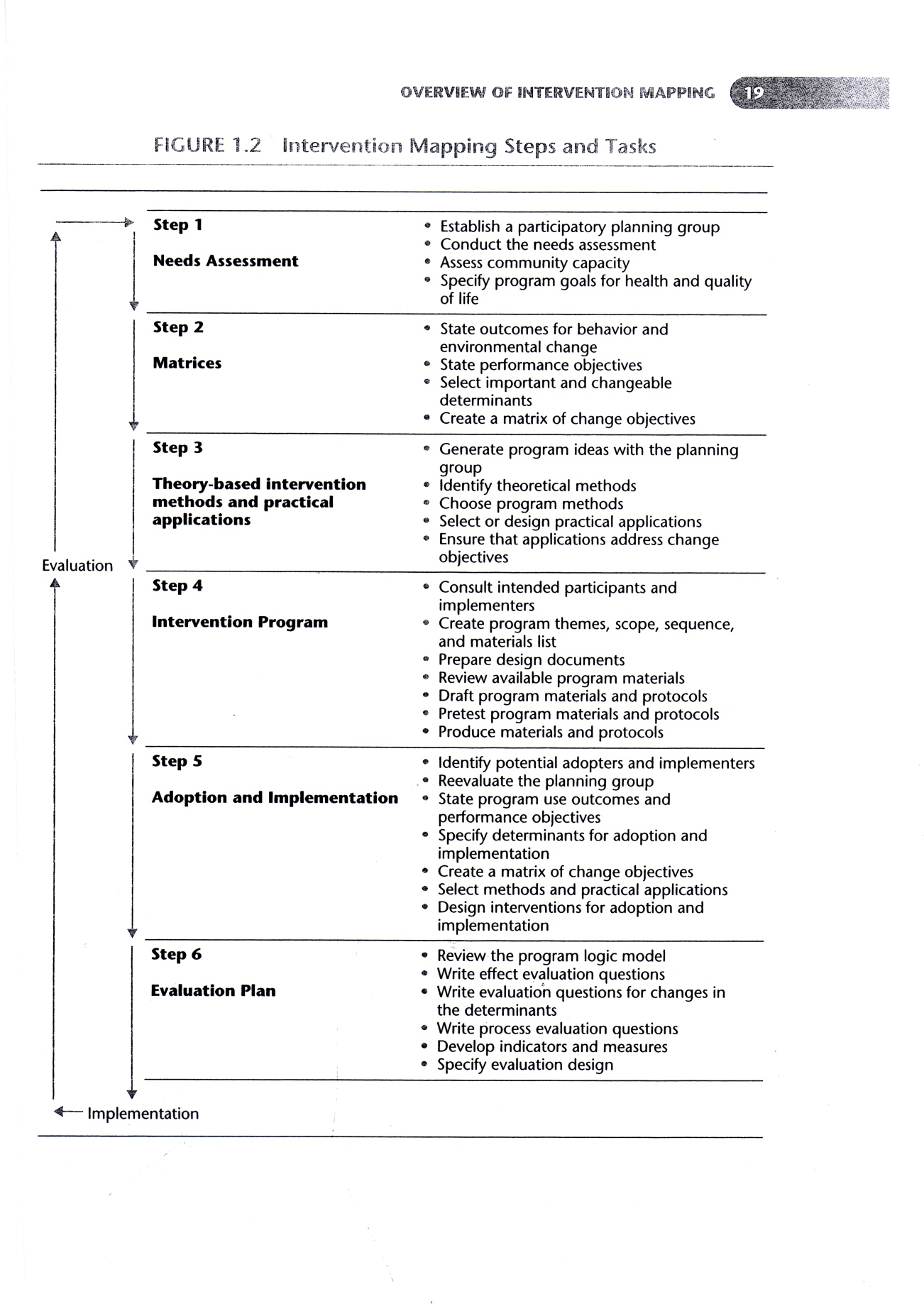


**Figuur 1. 1 Intervention Mapping (Bartholomew, Parcel, Kok & Gottlieb, 2011)**
